# Health care accessibility and mobility in breast cancer: a Latin American perspective

**DOI:** 10.1186/s12913-024-11222-6

**Published:** 2024-06-25

**Authors:** André Ferreira Azeredo-da-Silva, Bruna Stella Zanotto, Flavia Martins, Nádia Navarro, Rafaela Alencar, Clarissa Medeiros

**Affiliations:** 1 HTAnalyze Consultoria e Treinamento Ltda, Rua João Abbott, 109, Petrópolis, Porto Alegre, RS 90460-150 Brazil; 2 Produtos Roche Químicos e Farmacêuticos S/A, Rua Doutor Rubens Gomes Bueno, 691, Santo Amaro, São Paulo, SP 04730-000 Brazil

**Keywords:** Latin America, Breast cancer, Mobility, Brazil

## Abstract

**Background:**

Latin America (LATAM) encompasses a vast region with diverse populations. Despite publicly funded health care systems providing universal coverage, significant socioeconomic and ethno-racial disparities persist in health care access across the region. Breast cancer (BC) incidence and mortality rates in Brazil are comparable to those in other LATAM countries, supporting the relevance of Brazilian data, with Brazil’s health care policies and expenditures often serving as models for neighboring countries. We evaluated the impact of mobility on oncological outcomes in LATAM by analyzing studies of patients with BC reporting commuting routes or travel distances to receive treatment or diagnosis.

**Methods:**

We searched MEDLINE (PubMed), Embase, Cochrane CENTRAL, LILACS, and Google Scholar databases. Studies eligible for inclusion were randomized controlled trials and observational studies of patients with BC published in English, Portuguese, or Spanish and conducted in LATAM. The primary outcome was the impact of mobility or travel distance on oncological outcomes. Secondary outcomes included factors related to mobility barriers and access to health services. For studies meeting eligibility, relevant data were extracted using standardized forms. Risk of bias was assessed using the Newcastle-Ottawa Scale. Quantitative and qualitative evidence synthesis focused on estimating travel distances based on available data. Heterogeneity across distance traveled or travel time was addressed by converting reported travel time to kilometers traveled and estimating distances for unspecified locations.

**Results:**

Of 1142 records identified, 14 were included (12 from Brazil, 1 from Mexico, and 1 from Argentina). Meta-analysis revealed an average travel distance of 77.8 km (95% CI, 49.1-106.48) to access BC-related diagnostic or therapeutic resources. Nonetheless, this average fails to precisely encapsulate the distinct characteristics of each region, where notable variations persist in travel distance, ranging from 88 km in the South to 448 km in the North.

**Conclusion:**

The influence of mobility and travel distance on access to BC care is multifaceted and should consider the complex interplay of geographic barriers, sociodemographic factors, health system issues, and policy-related challenges. Further research is needed to comprehensively understand the variables impacting access to health services, particularly in LATAM countries, where the challenges women face during treatment remain understudied.

**Trial registration:**

CRD42023446936.

**Supplementary Information:**

The online version contains supplementary material available at 10.1186/s12913-024-11222-6.

## Background

Limited geographic access to health facilities is a major factor contributing to reduced utilization of health services, resulting in poorer health outcomes [1]. This issue is particularly critical in the context of patients with cancer, as their treatment typically involves a combination of surgery, radiotherapy, and/or chemotherapy, often requiring multiple visits to health facilities. Geographic barriers that impede access may delay treatment initiation, leading to suboptimal outcomes or even premature and preventable deaths. The burden of travel demands on patients with cancer has been linked to more advanced disease at diagnosis, flawed treatment, a grimmer prognosis, and diminished quality of life [2].

Brazil’s breast cancer (BC) incidence and mortality rates are comparable to those of other Latin American countries. For instance, the age-standardized incidence rates of BC per 100,000 women are 62.9 in Brazil, 61.1 in Argentina, and 49.6 in Mexico, illustrating that Brazil’s epidemiological data are within the regional range [3]. This epidemiological consistency supports the relevance of Brazilian data to the broader Latin American context. As Latin America’s largest economy, Brazil’s health care policies and expenditures influence regional trends and often serve as models for neighboring countries [4].

In Brazil, a country of continental dimensions, more than half of patients with cancer are required to travel from their hometown to another city to receive treatment, with persistent disparities in regional accessibility despite the shorter travel distances recently observed in some states [2, 5–7]. For example, there are 359 dedicated public treatment centers with asymmetric geographical distribution, where approximately 80% are located in 2 of the 5 Brazilian regions and 20% in the remaining regions [8, 9]. Patients with cancer who must commute for treatment face considerable challenges, including fatigue, long waiting times for their return trip, inadequate nourishment, financial constraints due to travel expenses, and disruption to daily life [10]. Radiotherapy and chemotherapy are of particular concern as they require frequent visits to cancer care facilities.

Low- and middle-income countries find themselves in diverse circumstances with respect to workforce capacity, regulation of private health care, public sector investment, care pathways, and the ineffectiveness of comprehensive national strategies for the development, management, sustainable financing, and accreditation of cancer care centers. Therefore, identifying issues of geographic mobility for patients with cancer in Latin America is important to ensure equitable access to care.

In this systematic review and meta-analysis, we aimed to evaluate the impact of mobility on oncological outcomes in Latin America by analyzing studies of patients with BC reporting their commuting routes or travel distances to receive treatment or diagnosis. We addressed 2 knowledge gaps: (1) whether BC treatment or screening programs have been made geographically accessible to patients in Latin American countries, and (2) whether the existing literature can provide regional estimates of travel distances to health facilities. The paper contributes to the worldwide debate on how to widen access to BC care and may pave the way for further developments and studies on the topic, while providing relevant data to the strategic planning of cancer care services.

## Methods

We developed this systematic review according to the PRISMA 2020 guidelines [11] and the recommendations proposed by the Cochrane Collaboration [12]. A detailed review protocol is available at PROSPERO (CRD42023446936).

### Search strategy

We searched MEDLINE (via PubMed), Embase, Cochrane Central Register of Controlled Trials (Cochrane CENTRAL), Latin American and Caribbean Health Sciences Literature (LILACS, via Virtual Health Library), and Google Scholar databases for articles published from inception to June 28, 2023, by entering the following keywords and terms individually, including index terms (MeSH and Emtree terms), subject indexes, and synonyms, or by combining them with Boolean operators (“AND” and “OR”): “Breast cancer,” “Breast neoplasm,” “Mobility,” “Access to healthcare,” and “Latin America.” Terms related to intervention or study design were not used to improve the search sensitivity. Although no language restrictions were imposed, we only considered articles published in English, Portuguese, or Spanish. We hand searched the reference lists of the included studies and of all reviews published to date on the topic to cover potential additional studies within the intended scope. The complete search strategy is provided in Additional Table 1. A cross-reference check to locate and eliminate duplicates complemented the search strategy.

**Table 1:** Main characteristics of the included studies

Author/year	Country (city)	Study design	Participants	Sample size	Age group^a^	Context	Outcome
Agudelo Botero et al. (2013)[23]	Mexico	Ecological study (ENSAR, ENSA, ENSANUT)	Women undergoing breast cancer screening	ENSAR: *n* = 11,800 ENSA: *n* = 21,338 ENSANUT: *n* = 10,182	NR	Breast cancer screening	Effect of type of area on access to cancer screening. Did not report distance or time traveled
Aguiar et al. (2023)[22]	Brazil (São Paulo)	Cross-sectional study	Breast cancer death records of women from 2009 to 2016	10,066	20 years or more	Breast cancer mortality	Time traveled
de Almeida et al. (2022)[20]	Brazil (São Paulo)	Cross-sectional study	Women with invasive breast cancer	81,669	56.8 years (SD 13.6)	Breast cancer diagnosis	Time traveled
de Souza et al. (2020)[18]	Brazil	Ecological study (DATASUS)	Women with breast cancer	36,137 HAAs*	NR	Breast cancer treatment	Variation in mean travel distance
Ferreira et al. (2020)[26]	Brazil	Ecological study (HRC, INCA)	Women with breast cancer	15,931	NR	Breast cancer treatment	Effect of the region on time from diagnosis to treatment
Oliveira et al. (2011)[7]	Brazil	Ecological study	Women with breast cancer	NR	NR	Breast cancer treatment	Travel distance
Saldanha et al. (2019)[6]	Brazil	Ecological study (DATASUS 2014–2016)	Women with breast cancer	117,841 HAAs*	NR	Breast cancer treatment	Travel distance
Medeiros et al. (2021)[21]	Brazil	Prospective cohort study	Women with breast cancer	470	56 (IQR 47–65)	Breast cancer treatment	Travel time
Recondo et al. (2019)[27]	Argentina (Buenos Aires)	Prospective cohort study	Women with breast cancer	168	58 (SD 13.4)	Breast cancer treatment	Effect of type of hospital on access to cancer treatment
Rodrigues et al. (2019)[24]	Brazil	Ecological study	Women undergoing breast cancer screening	NR	NR	Breast cancer screening	Effect of area on cancer screening
Romeiro Lopes et al. (2017)[28]	Brazil	Cross-sectional study	Women with breast cancer	82	NR	Breast cancer treatment	Effect of area on cancer screening. Did not report travel time or distance
Gonçalves et al. (2014)[29]	Brazil	Cross-sectional study	Women with breast cancer	16	53.5	Breast cancer treatment	Effect of transferring or transport on cancer treatment. Did not report travel time or distance
Sousa et al. (2019)[19]	Brazil	Cross-sectional study	Women with breast cancer	155	53.6 (SD 12.4)	Breast cancer treatment	Percentage of delay (> 60 days)
Amaral et al. (2017)[25]	Brazil	Cross-sectional study	Women undergoing breast cancer screening	NR	NR	Breast cancer screening	Mammograms not performed because of distance (> 60 km)

DATASUS indicates Information Technology Department of the Brazilian Unified Health System; ENSAR, *Encuesta Nacional de Salud Reproductiva* (National Reproductive Health Survey); ENSA, *Encuesta Nacional de Salud* (National Health Survey); ENSANUT, *Encuesta Nacional de Salud y Nutrición* (National Health and Nutrition Survey); HAA, hospital admission authorization; HRC, Hospital Registry of Cancer; INCA, Brazilian National Cancer Institute; IQR, interquartile range; NR, not reported; SD, standard deviation

^a^(years, median/mean/range) *It refers to admission to qualified hospitals for clinical and surgical procedures, and each HAA represents the total number of hospital admissions rather than the number of patients

### Eligibility criteria and study selection

Studies eligible for inclusion in this review were published in English, Portuguese, or Spanish and recruited patients with BC in Latin American countries. The study designs considered for inclusion were randomized controlled trials and observational studies (cohort, cross-sectional, case control, case series, or ecological studies) with or without a comparison group, regardless of the intervention used. We excluded conference abstracts, guidelines, editorials, book chapters, commentaries, letters, notes, and study protocols.

We limited the scope of the review to Latin America because we intended to explore mobility-related factors alongside health care resource utilization in Latin American populations. Furthermore, this decision stemmed from the shared health patterns observed in Latin American countries, characterized by popular-collective health care, recurrent discontinuity in public policies—an inherent feature of the region—and a prevailing culture of prioritizing urgency in professional endeavors.

Studies were considered for inclusion if they clearly reported the travel distance (in kilometers or other units) or time (in hours, minutes) required to access BC-related health care. The primary outcome of this review was the impact of mobility or travel distance on oncological outcomes such as mortality, time to treatment initiation, and time to diagnosis. Secondary outcomes included mobility-related factors such as geographic barriers, access to municipal transportation, and travel time.

### Data extraction process

After removal of duplicates, 2 reviewers (AFA and BSZ) independently screened titles and abstracts, and then screened potentially eligible or candidate full-text articles for selection based on the inclusion and exclusion criteria. A third independent reviewer was consulted to settle any disagreements between reviewers that had not been resolved by consensus. From studies of overlapping populations, we included only the one with the largest sample size.

The same 2 reviewers (AFA and BSZ) independently extracted data from eligible studies using a standardized form. Disagreements were resolved with discussion and, if required, consensus was reached by consulting a third independent reviewer. The following data were extracted: study characteristics (e.g., author, year, study setting, study design, and study context), sample characteristics (e.g., number of participants, age of participants, and sample size), characteristics of the tools used to measure mobility or access, and comparison groups (if available).

### Risk of bias assessment

The same reviewers (AFA and BSZ) independently assessed the risk of bias of each included study. The original Newcastle-Ottawa Scale (NOS) was used to assess cohort studies comparing treatment options. It consists of 8 items that classify methodological quality across 3 categories by a star rating system: participant selection (maximum 4 stars), comparability (maximum 2 stars), and assessment of outcome (maximum 3 stars) [13]. In the NOS adapted for cross-sectional studies, a maximum of 10 stars can be awarded to each study: selection (maximum 5 stars), comparability (maximum 2 stars), and outcome (maximum 3 stars). Studies reaching 75% or more of the maximum number of stars are considered to be at low risk of bias, while those reaching 50–75% are considered to be at moderate risk of bias.

### Data analysis and travel distance estimates

We performed a synthesis of qualitative and quantitative evidence. We collected data on the main findings and consequences of mobility as assessed in each study and the related oncological outcomes. Given the heterogeneity among study results regarding distance traveled or travel time, we decided to use the distance traveled instead of travel time given the relatively deficient and expensive transport systems in Latin American countries. When the outcomes were reported in travel time instead of distance traveled, the study authors were contacted. If there was no response or the data were unavailable, a conversion technique was used. To address this issue, when a study reported data on travel time, we converted the data to kilometers traveled based on the estimates provided by INRIX (vehicle monitoring and software company), which assumes an average city traffic of 30.2 km/h [14, 15]. If necessary, we used WebPlotDigitalizer [16] to extract data from figures and graphs. For studies reporting parameters such as ‘outside the city,’ we estimated the average distance from the capital city to nearby cities using Google Maps.

Based on the data presented in the included studies, we estimated the average distance traveled by people to access BC screening services and by patients with BC to access treatment facilities. Since most studies did not provide sufficient data to estimate the standard error of the distance traveled, we calculated standard errors for the set of average distances estimated for the different studies (imputation-driven meta-analysis). We attributed the standard errors to all studies so that all of them had the same weight in the meta-analysis, as calculated using the inverse variance method. We used R software (meta package v 6.0–0) for data analysis [17].

## Results

### Study selection and included studies

**Fig. 1:**
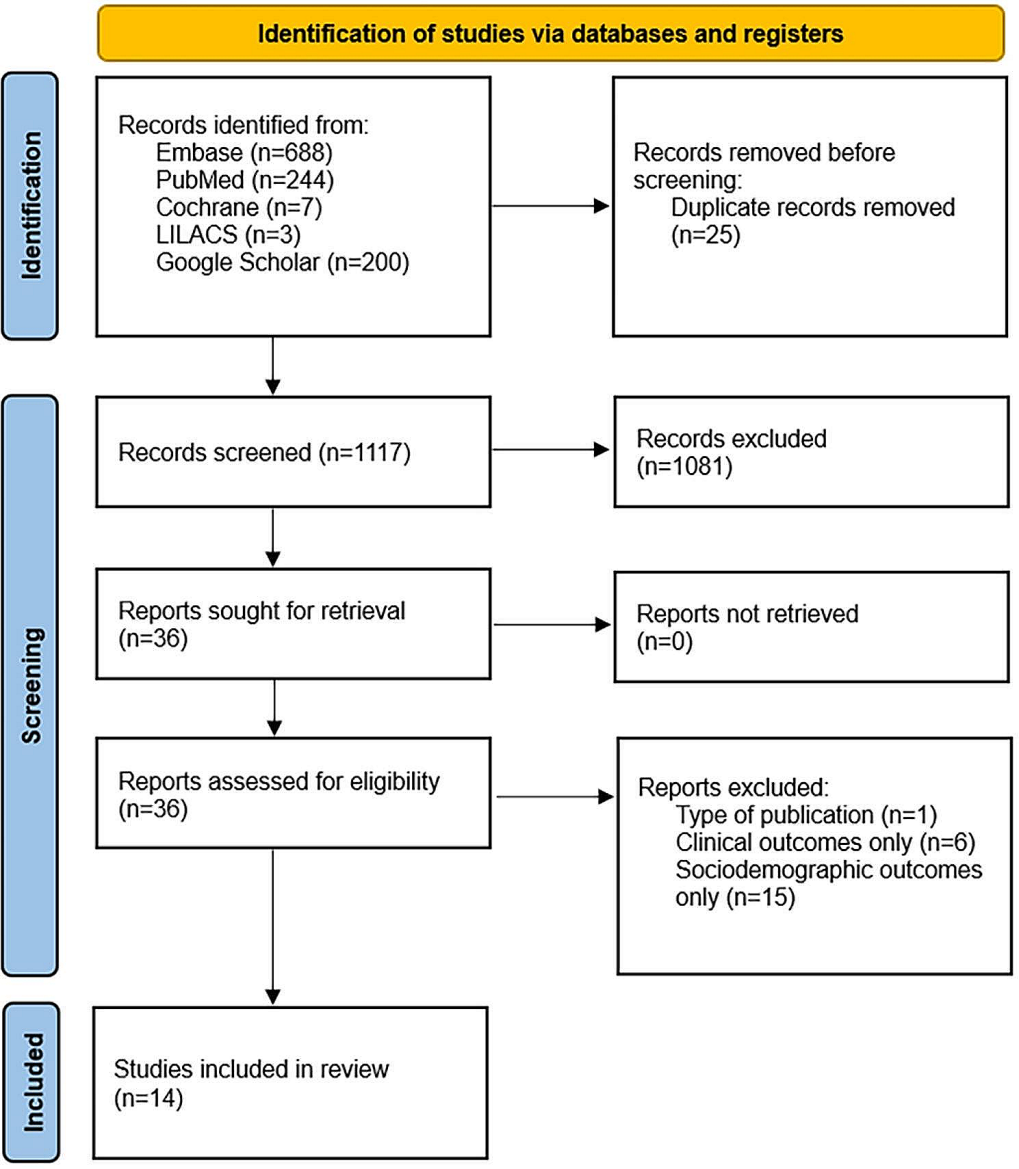
PRISMA flow diagram

The study selection process is shown in Fig. 1. The database searches provided a total of 1142 records. After adjusting for duplicates, 1117 remained. After title and abstract screening, a total of 36 studies were retrieved for full-text review, 14 of which met the inclusion criteria.

### Characteristics of included studies

Table 1 provides the main characteristics of each included study, an outline of the individual characteristics of each study population, and the context in which the impact of mobility was studied. Regarding mobility outcomes, 4 studies reported travel distance [6, 7, 18, 19], 3 studies reported travel time [20–22], and 7 studies reported any effect measure such as mammograms not performed or effect on access to cancer screening [23–29]. To facilitate understanding of the results, the following sections are divided into the impact of mobility on BC screening and the impact of mobility on BC treatment.

### Impact of mobility on BC screening

Three studies evaluated BC screening. The study of Agudelo Botero et al. [23] used secondary data from 3 Mexican databases to explain factors that can impact BC screening for women. In all databases, the sociodemographic variables that together could explain the strongest relationship with breast self-examination were level of education, age group, and type of area (urban vs. rural). The other 2 studies were conducted in Brazil by Rodrigues et al. [24] and Amaral et al. [25] and found results similar to those of the Mexican study. Rodrigues et al. [24] reported that the spatial coverage of mammography machines, using 60 km as a parameter for the maximum distance between an individual’s home and a mammography machine, was fully achieved in the South and Southeast regions and several states in the Northeast but not in the North and Midwest regions.

### Impact of mobility on BC treatment

Several studies investigated the association between travel burden and BC treatment. de Almeida et al. [20] reported an increased likelihood of advanced BC stage at diagnosis in patients who traveled to another city for BC care. Ferreira et al. [26] evaluated over 150,000 women with BC and concluded that those categorized as non-white with a low level of education living in the North of Brazil had to wait longer from diagnosis to treatment than women in other groups, in addition to being more likely to wait over 60 days to start BC treatment. Oliveira et al. [7] highlighted that a high percentage of women receiving treatment through the Brazilian publicly funded health care system lived more than 150 km away from the place of care. Knowing that treatment is based on frequent procedures, the authors noted that a large proportion of women receiving care had to face a number of difficulties other than the disease itself due to long travel distances.

Saldanha et al. [6] reported that commuting can affect 51.34% of BC patients negatively, with over half requiring journeys of more than 3 h in half of the cases. The proportion of patients who need to travel outside their hometown for chemotherapy and radiotherapy is similar to that for hospital admissions. However, given that these therapies require multiple visits to health facilities during the treatment cycle, their potential impact on the quality of life of women undergoing treatment is of particular concern. In a prospective study conducted in Brazil, Medeiros et al. [21] showed that living outside the city of Rio de Janeiro and older age were associated with a time interval between diagnosis and treatment initiation exceeding 60 days, despite the ‘60-day law’ in place since 2013 establishing that treatment for any type of cancer in the public health system must start within 60 days of the diagnosis. In a study comparing private and public hospitals in the city of Buenos Aires, Argentina, regarding BC treatment, Recondo et al. [27] found that patients receiving treatment in public hospitals used public transport more often (69.3%) than those treated in private hospitals (29.3%), resulting in significantly longer commutes for those treated in public hospitals. In southern Brazil, Romeiro Lopes et al. [28] found a mean time to diagnosis of 102.5 (SD 165.5) days, with treatment delay in 63.4% (*n* = 52) of cases, where 60% of patients with a delay in treatment > 30 days lived more than 100 km from the cancer care center. Although without statistical significance, this finding draws attention as a factor influencing treatment adherence over time.

Unlike the previous findings, 2 studies [24, 29] did not report geographic distance or commuting as the main access barriers. Evaluating barriers to access to health care as perceived by women with BC in northeastern Brazil, Gonçalves et al. [29] reported that geographic barriers were rarely mentioned by women during treatment, but this factor requires attention because transfer to another city and difficulty accessing transport provided by the municipal health department were mentioned by the participants, capturing the reality of the Northeast region. Also in the Northeast of Brazil, de Sousa et al. [18] demonstrated that, despite important data on geographic distance and time to treatment, treatment delay was not linked to geographic barriers but rather to a fragmentation of health services, that is, to a need to shift the points of care from primary to specialized care with a well-defined patient flow. Finally, Aguiar et al. [22] reported that work commutes of 1 to 2 h were negatively associated with BC mortality in the city of São Paulo, Brazil, and that these findings were important to guide cancer prevention policies.

### Quantitative data analysis

Table 2 provides the quantitative results of individual studies and the impact of mobility on the related oncological outcomes. Regarding quantitative data analysis, the heterogeneity was notably high (I^2^ = 93%), and the number of studies that provided sufficient information for a meta-analysis was limited to 7, rendering the meta-analysis inadequate for reporting the primary outcome [6, 7, 19, 20, 22, 26]. We performed an exploratory subgroup analysis to investigate the regions of Brazil where the studies had been conducted as a potential source of heterogeneity. Indeed, this analysis revealed that a portion of the observed heterogeneity stemmed from variations in the regions where the studies had been conducted.

**Table 2:** Quantitative study results

Author/year	Context and study design	Category 1 (C1)	Category 2 (C2)	Outcome reported in the study	Outcome value reported in the study	Distance outcome (km) (converted if necessary)	Measure of association
Agudelo Botero et al. (2013)[23]	Breast cancer screening (ecological study)	Rural (*N* = 2984, ENSAR) Rural (*N* = 10,025, ENSA) Rural (*N* = 2512, ENSAR) Rural (*N* = 3169)	Urban (*N* = 8816, ENSAR) Urban (*N* = 11,363, ENSA) Urban (*N* = 7670, ENSAR) Urban (*N* = 9112, ENSANUT)	Effect of type of area on access to cancer screening	C1 = 0.72, C2 = 0.79 C1 = 0.12, C2 = 0.13 C1 = 0.44, C2 = 0.47 C1 = 1.8, C2 = 1.8	NR	RR = 0.91 RR = 0.92 RR = 0.94 RR = 1
**Aguiar et al. (2023)**[22]	**Breast cancer mortality (cross-sectional study)**	**Work commute < 1 h**	**Work commute of 1 to 2 h**	**Work commute time**	**1 to 2 h**	**30 to 60 km**	**RR 0.97** **(0.93-1)**
**de Almeida et al. (2022)**[20]	**Breast cancer diagnosis at advanced stage (cross-sectional study)**	**Advanced at the time of diagnosis (** *N* ** = 38,619)**	**Not advanced at the time of diagnosis (** *N* ** = 15,847)**	**Travel for cancer care**	**3 h**	**100 km**	**OR 1.07** **(1.04–1.10)**
de Souza et al. (2020)[18]	Breast cancer treatment (ecological study)	Year 2004 North = 1736 km Northeast = 1734 km Southeast = 1003 km South = 1105 km Midwest = 1226 km	Year 2010 North = 1736 km Northeast = 1734 km Southeast = 1127 km South = 1633 km Midwest = 1226 km	Variation in mean travel distance to treatment	North = 0 Northeast = 0 Southeast = 124 km South = 528 km Midwest = 0	North = 0 Northeast = 0 Southeast = 124 km South = 528 km Midwest = 0	NR
Ferreira et al. (2020)[26]	Breast cancer treatment (ecological study)	North North North North	Northeast Midwest Southeast South	Time from diagnosis to treatment > 60 days	NR	NR	OR 0.53 (0.5–0.57) OR 0.25 (0.32–0.38) OR 0.71 (0.66–0.76) OR 0.8 (0.75–0.85)
Oliveira et al. (2011)[7]	Breast cancer treatment (ecological study)	NR	NR	Travel distance	Surgery = 67 km Chemotherapy = 108 km Radiotherapy = 94 km	Surgery = 67 km Chemotherapy = 108 km Radiotherapy = 94 km	NR
Saldanha et al. (2019)[6]	Breast cancer treatment (ecological study)	NR	NR	Median travel distance	North = 448 km Northeast = 147 km Midwest = 323 km Southeast = 117 km South = 88 km	North = 448 km Northeast = 147 km Midwest = 323 km Southeast = 117 km South = 88 km	NR
				**Mean travel time from city of residence to care center**	**3 h**	**180 km**	
Medeiros et al. (2020)[21]	Breast cancer treatment (prospective cohort study)	Living in Rio de Janeiro city (*N* = 273)	Living outside Rio de Janeiro city (*N* = 197)	Time from diagnosis to treatment > 60 days	NR	Median travel distance 142.5 km	OR 2.57 (1.31–5.05)
Recondo et al. (2019)^27^	Breast cancer treatment (prospective cohort study)	Private hospitals (*N* = 93)	Public hospitals (*N* = 75)	Time from diagnosis to chemotherapy	C1 = 60 days, C2 = 76 days C1 = 0.6 h, C2 = 0.8 h	C1 = 11 km (6–10) C2 = 13 km (5.1–30)	NR
Rodrigues et al. (2019)[24]	Breast cancer screening (ecological study)	Mammograms available for women aged 50 to 69 years	Mammograms not available for women aged 50 to 69 years	Within 60 km	North = 70.2% Northeast = 89.9% Southeast = 86.6% South = 98.1% Midwest = 95.3%	60 km	NR
Romeiro Lopes et al. (2017)[28]	Breast cancer treatment (cross-sectional study)	Cities ≤ 100 km (*N* = 19)	Cities > 100 km (*N* = 15)	Time from diagnosis to treatment	C1 = 12; C2 = 9	NR	OR 1.49 (0.23–7.09)
		Living in the city (*N* = 48)	Outside the city (*N* = 34)		C1 = 31; C2 = 21	NR	OR 1.46 (0.3–7.01)
Gonçalves et al. (2014)[29]	Breast cancer treatment (cross-sectional study)	NR	NR	Transfer to another city	2 (7.7%)	NR	NR
				Difficulty accessing transport provided by municipal health dept	2 (7.7%)		
**Sousa et al. (2019)**[19]	**Breast cancer treatment (cross-sectional study)**	**Cocais** **Serra da Capivara** **Carnaubais** **Vale do Canindé** **Chapada das Mangabeiras**	**Entre Rios** **Entre Rios** **Entre Rios** **Entre Rios** **Entre Rios**	**Percentage of delays between diagnosis and chemotherapy (> 60 days)**	**92.9%** **85.7%** **80.0%** **80.0%** **66.7%**	**166.9 km** **521.6 km** **84 km** **278.9 km** **603 km**	**NR**
Amaral et al. (2017)[25]	Breast cancer screening (cross-sectional study)	NR	NR	Mammograms not performed because of distance (> 60 km)	2.1 million	60 km	1 in 5.8 expected mammograms

ENSA indicates *Encuesta Nacional de Salud* (National Health Survey); ENSAR, *Encuesta Nacional de Salud Reproductiva* (National Reproductive Health Survey); ENSANUT, *Encuesta Nacional de Salud y Nutrición* (National Health and Nutrition Survey); NR, not reported; OR, odds ratio; RR, relative risk. Bold indicates the studies whose measures were converted for quantitative analysis. Whenever a measure of association was used in the studies, C2 was used as a reference

**Fig. 2:**
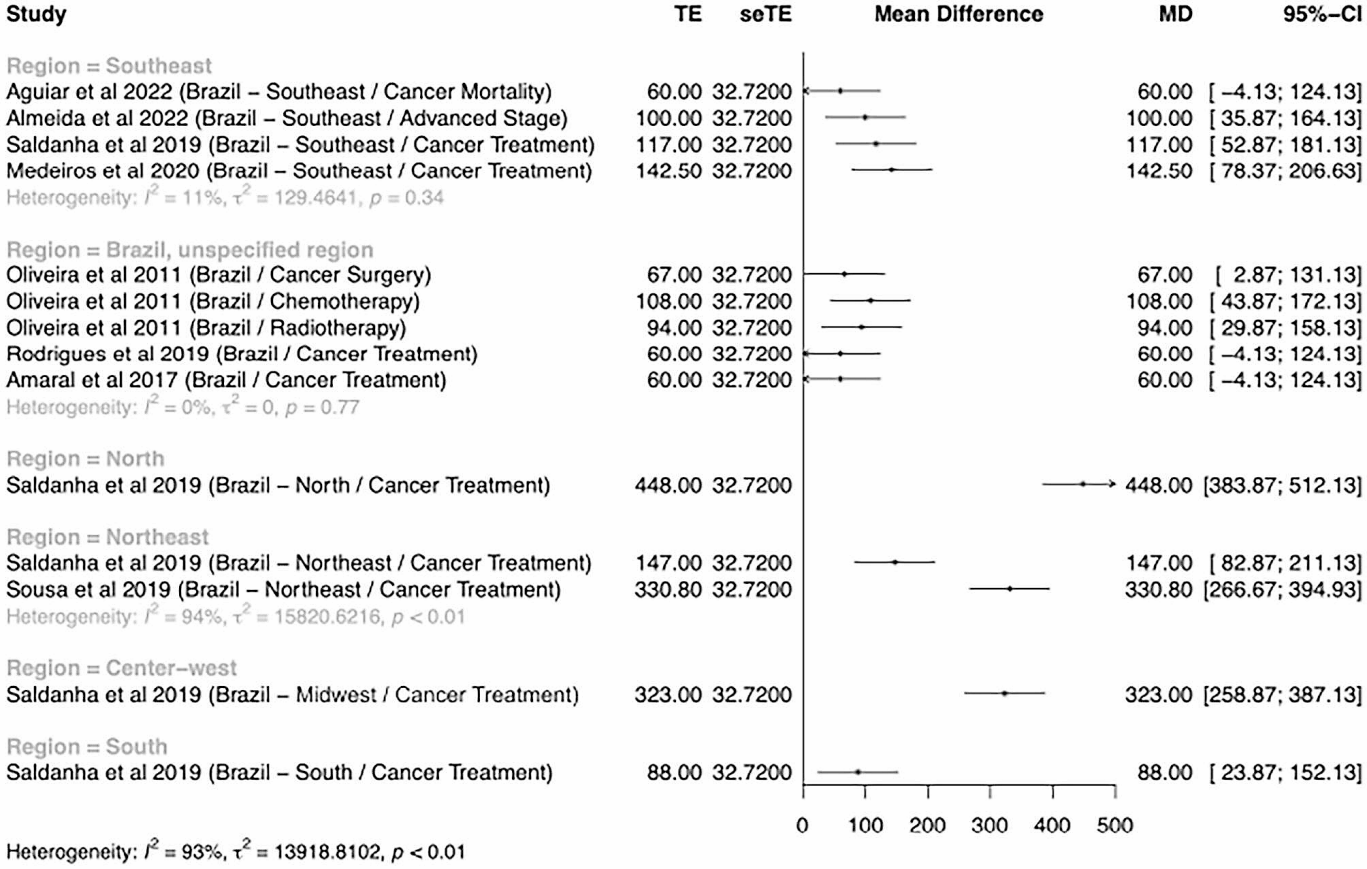
Forest plot of average travel distances reported in the studies

The average distances traveled are shown in the forest plot in Fig. 2. For hypothesis generation purposes only, the average distances traveled to BC-related diagnostic or therapeutic resources in the 5 administrative regions of Brazil were estimated via a random-effects meta-analysis (Additional Fig. 1), yielding the following results: 448 km (95% CI, 383.87–512.13) in the North; 323 km (95% CI, 258.87–387.13) in the Midwest; 239.8 km (95% CI, 58.78–419.02) in the Northeast; 104.8 km (95% CI, 70.93–138.82) in the Southeast; and 88 km (95% CI, 23.87–152.13) in the South. Four studies reported results for Brazil as a whole, without specifying a region. For description purposes, these results indicate an average travel distance of 77.8 km (95% CI, 49.1–106.48) to a BC-related diagnostic or therapeutic resource. Even though we acknowledge the limitations and regional disparities both within and between countries, our findings align with the existing literature, indicating an equivalent of 3–4 h of commute on a national average [30].

### Risk of bias assessment

Overall, the risk of bias was moderate to low. The 12 cross-sectional non-comparative studies were rated with a median of 7.5 stars on the adapted NOS (maximum 10 stars). The 2 cohort studies were also rated as having a moderate to low risk of bias (7 and 8 out of 9 stars, respectively) on the original NOS (Additional Table 2). Particularly in this analysis, the risk of bias had an impact on the interpretability of the studies.

## Discussion

Access to and affordability of appropriate diagnosis and care represent critical limiting factors in health care [31]. The establishment of national BC plans, whether of a general or specific nature, plays a pivotal role in facilitating organized governance, financing, and health care delivery [20, 31]. In this regard, evidence-based treatment guidelines have been disseminated by government authorities, cancer institutes, or scientific associations in numerous countries. Nevertheless, the principal challenge lies in the effective implementation of policies and mechanisms designed to ensure consistent compliance with these guidelines over the entire population.

Consistent with the existing literature, our research findings underscore the presence of regional disparities across the health care landscape of Brazil [31]. Specifically, our analysis revealed that patients living in the North and Midwest of the country must travel longer distances to access cancer care than their counterparts in the South, Southeast, and Northeast [30]. It is worth noting that, despite the existence of Law No. 12,732, which mandates a 60-day time frame for initiating cancer treatment after the disease has been diagnosed, there is a lack of empirical evidence to define what constitutes a reasonable travel distance for such treatment [32], since approximately 40% of patients experience a delay in starting their treatment of more than 60 days, and this delay is longer in the SUS than in the private health insurance system [26, 33]. In other words, the law addresses the number of days for initiating treatment but does not establish what distance is considered to be reasonable for patients to obtain such treatment. de Almeida et al. [20] showed that women traveling to another city to receive BC care were more likely to have advanced disease at the time of diagnosis and that late diagnosis increases the cost of treatment and compromises the patient’s clinical outcome. Despite the ‘60-day law’ and health care policy initiatives in Brazil, there appears to be a gap between policy intentions and their actual implementation, particularly for patients living outside major urban centers [22, 23, 27, 29]. Some studies have highlighted the underrepresentation of geographic barriers in patients’ perceptions, emphasizing the need for a nuanced contextual understanding.

In the context of BC screening, the health care system should be designed to ensure an adequate number of mammography machines, with due consideration for a maximum distance of 60 km between the machine and the residences of the target population [25, 34]. However, although this spatial proximity is deemed essential to facilitate timely and accessible screening services for BC detection, women continue to face difficulties in accessing appropriate screening, and by the time they do, they often present at an advanced disease stage [20]. Nonetheless, in addition to distance, Bretas et al. [35] pointed out the lack of a well-defined strategy to receive women with self-detected breast abnormalities in the primary health care unit. Strategies may encompass procedures such as enhancement of clinical breast examination, breast biopsy, and accurate pathology as well as BC surveillance and telehealth. Such actions take place occasionally in one-stop clinics, although patients will often be transferred to different locations [35]. Therefore, patient navigation programs and integration between primary and tertiary care need to be further improved.

Long travel distances to radiotherapy centers have been associated with diminished utilization of radiotherapy services, elevated mastectomy rates in patients with BC, reduced probability of radiotherapy utilization among individuals with BC and other cancers, and infrequent recourse to palliative radiotherapy [30]. While it is important to acknowledge that patient travel distance is not the only determinant of access to cancer services, it remains a pivotal factor to be addressed in endeavors to enhance health equity and achieve a broader health coverage [7].

The concentration of specialized cancer care to centers located in the Southeast of Brazil highlights the need to narrow the gap between supply and demand for this type of care. Providing broad coverage of cancer treatment requires improved planning and regulation, in addition to ensuring the activation of the highly complex infrastructure and qualified human resources that are needed to support treatment [30, 31].

The obstacles to mobility and access to BC screening and treatment identified in Brazil, such as geographic barriers, socioeconomic inequalities, and health care infrastructure limitations, resonate with challenges faced by other Latin American countries. Practical solutions to overcome these barriers include implementing telemedicine services and mobile health units [36] and expanding the role of community health workers to provide education, support, and navigation services [37]. Policymakers can leverage Brazil’s experiences to inform regional strategies, such as the Brazilian National Policy of Comprehensive Women’s Health Care, which provides a framework for addressing women’s health issues, including BC, adaptable by other Latin American countries to improve outcomes [38].

### Limitations

The heterogeneity of the studies renders the meta-analytic estimates not representative of the overall travel distances observed in the included studies. Even though our objective was to conduct a comprehensive literature review within a Latin American perspective, there were only 2 studies outside Brazil. It was expected that Brazil, Mexico, and Argentina, the largest Latin American countries, would be better represented in the literature, but the lack of studies from other countries in Latin America makes generalization difficult. While concerns about the regional representativeness of Brazilian studies are valid, the similarities in demographics, socioeconomic status, health care structures, and epidemiological trends across Latin America support the relevance of Brazilian data. By addressing practical implications and proposing evidence-based solutions, we aim to improve BC screening and treatment accessibility throughout the region. Besides that, the fact that a sensitive search strategy was unable to retrieve studies from a more diverse group of countries shows a wide gap in the scientific literature on this topic in other countries in Latin America. Furthermore, our analyses and results were limited by the need to convert travel time to travel distance when data were not available even after contacting authors, which could have underestimated or overestimated some results especially in remote areas where the transportation infrastructure is poor. However, this approach allowed the comparison of travel distances in diverse settings.

## Conclusions

The collective evidence from these studies underscores the multifaceted and pervasive influence of mobility and travel distance on access to BC care. It also emphasizes the importance of not only addressing geographic barriers but also considering sociodemographic factors, health system issues, and policy-related challenges in the pursuit of equitable BC care. The scarce information on this topic in Latin American countries, especially on the complications and challenges women face before and during treatment, indicates that travel distance alone may not serve as the only determinant of mobility. Therefore, additional research is imperative to comprehensively elucidate the multifaceted variables that underlie the impact of mobility on access to health services.

### Electronic supplementary material

Below is the link to the electronic supplementary material.

Supplementary Material 1

Supplementary Material 2. **Additional** Fig. 1. Meta-analysis forest plot

Supplementary Material 3

## Data Availability

All data analyzed during this study are included in this published article and its supplementary information files.

## Abbreviations

BC: breast cancer
NOS: Newcastle-Ottawa Scale
SUS: Brazilian national public health care system
