# Supplementary material for: Health care accessibility and mobility in breast cancer: a Latin American perspective
Source: BMC Health Serv Res. 2024 Jun 25;24:764. doi: 10.1186/s12913-024-11222-6 (PMC11197349; doi:10.1186/s12913-024-11222-6)
Supplement: Supplementary file 1 — Supplementary Material 1 [file 12913_2024_11222_MOESM1_ESM.docx]

**Additional Table 1.** Search strategy and number of hits per database

| **Base** | **Query** | **Date** | **Hits** |
| --- | --- | --- | --- |
| Embase | ('breast cancer'/exp OR 'breast gland cancer' OR 'breast gland neoplasm' OR 'breast malignancies' OR 'breast malignancy' OR 'breast tumor malignant' OR 'ca breast' OR 'cancer in the mammary gland' OR 'cancer of the breast' OR 'cancer of the mammary gland' OR 'cancer, breast' OR 'malignancies of the breast' OR 'malignancy of the breast' OR 'malignant breast neoplasm' OR 'malignant breast tumor' OR 'malignant neoplasm of the breast' OR 'malignant tumor of the breast' OR 'mamma cancer' OR 'mammary cancer' OR 'mammary gland cancer' OR 'mammary gland malignancy' OR 'mammary malignancies' OR 'mammary malignancy' OR 'breast cancer') AND (('access to health care'/exp OR 'access to health care' OR 'health services accessibility'/exp OR 'health services accessibility' OR 'healthcare access'/exp OR 'healthcare access' OR 'health care access'/exp OR 'health care access' OR 'health system delay') AND ('health care access'/exp OR 'health care access') OR 'access to care'/exp OR 'access to care' OR 'patient mobility'/exp OR 'patient mobility' OR 'mobility'/exp OR mobility OR 'travel'/exp OR travel OR 'relocation'/exp OR relocation OR 'migration'/exp OR migration) AND ('brazil'/exp OR 'brazil' OR 'brazilian'/exp OR 'brazilian' OR 'south and central america'/exp OR 'mexico'/exp) AND [embase]/lim | 06/28/2023 | 688 |
| MEDLINE (via PubMed) | ('breast cancer'/exp OR 'breast gland cancer' OR 'breast gland neoplasm' OR 'breast malignancies' OR 'breast malignancy' OR 'breast tumor malignant' OR 'ca breast' OR 'cancer in the mammary gland' OR 'cancer of the breast' OR 'cancer of the mammary gland' OR 'cancer, breast' OR 'malignancies of the breast' OR 'malignancy of the breast' OR 'malignant breast neoplasm' OR 'malignant breast tumor' OR 'malignant neoplasm of the breast' OR 'malignant tumor of the breast' OR 'mamma cancer' OR 'mammary cancer' OR 'mammary gland cancer' OR 'mammary gland malignancy' OR 'mammary malignancies' OR 'mammary malignancy' OR 'breast cancer') AND (('access to health care'/exp OR 'access to health care' OR 'health services accessibility'/exp OR 'health services accessibility' OR 'healthcare access'/exp OR 'healthcare access' OR 'health care access'/exp OR 'health care access' OR 'health system delay') AND ('health care access'/exp OR 'health care access') OR 'access to care'/exp OR 'access to care' OR 'patient mobility'/exp OR 'patient mobility' OR 'mobility'/exp OR mobility OR 'travel'/exp OR travel OR 'relocation'/exp OR relocation OR 'migration'/exp OR migration) AND ("Latin America"[MeSH Terms] OR "Brazil"[MeSH Terms] OR "Mexico"[MeSH Terms] OR "Central America"[MeSH Terms] OR "South America"[MeSH Terms]) | 06/28/2023 | 244 |
| Cochrane CENTRAL | ID Search Hits  #1 MeSH descriptor: [Breast Neoplasms] explode all trees 17781  #2 MeSH descriptor: [Latin America] explode all trees 169  #3 MeSH descriptor: [South America] explode all trees 3866  #4 MeSH descriptor: [Central America] explode all trees 372  #5 MeSH descriptor: [Brazil] explode all trees 2325  #6 MeSH descriptor: [Mexico] explode all trees 910  #7 MeSH descriptor: [Delivery of Health Care] explode all trees 61093  #8 ('access to health care' OR 'access to health care' OR 'health services accessibility' OR 'health services accessibility' OR 'healthcare access' OR 'healthcare access' OR 'health care access' OR 'health care access' OR 'health system delay') AND ('health care access' OR 'health care access') OR 'access to care' OR 'access to care' OR 'patient mobility' OR 'patient mobility' OR 'mobility' OR mobility OR 'travel' OR travel OR 'relocation' OR relocation OR 'migration' OR migration 43932  #9 #2 OR #3 OR #4 OR #5 OR #6 5171  #10 #7 OR #8 101568  #11 #1 AND #9 AND #10 7 | 06/28/2023 | 7 |
| LILACS | mh:("Acesso aos Serviços de Saúde" OR "Barreiras ao Acesso aos Cuidados de Saúde") AND ("Neoplasias da Mama") AND ("América Latina")  mh:("Access to Health Services" OR "Barriers to Access to Health Care") AND ("Breast Neoplasms") AND ("Latin America")  mh:("Acceso a los Servicios de Salud" O "Barreras al Acceso a la Atención de la Salud") AND ("Neoplasias de la Mama") AND ("América Latina") | 06/28/2023 | 3 |
