# Supplementary figures and images for: Health care accessibility and mobility in breast cancer: a Latin American perspective
Source: BMC Health Serv Res. 2024 Jun 25;24:764. doi: 10.1186/s12913-024-11222-6 (PMC11197349; doi:10.1186/s12913-024-11222-6)

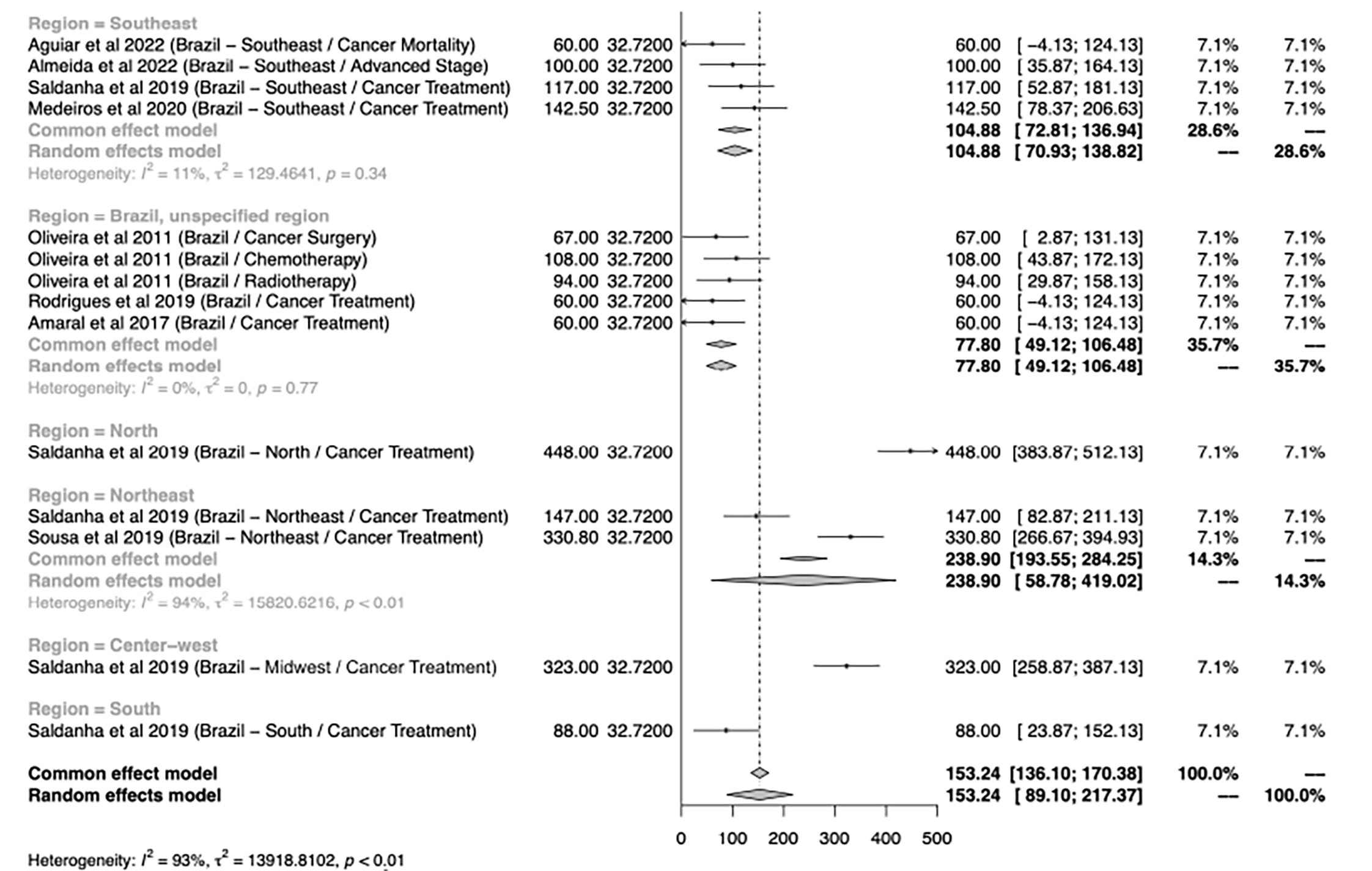

Supplement: Supplementary file 2 — Supplementary Material 2. Additional Fig. 1. Meta-analysis forest plot [file 12913_2024_11222_MOESM2_ESM.jpg]
