# Supplementary material for: Health care accessibility and mobility in breast cancer: a Latin American perspective
Source: BMC Health Serv Res. 2024 Jun 25;24:764. doi: 10.1186/s12913-024-11222-6 (PMC11197349; doi:10.1186/s12913-024-11222-6)
Supplement: Supplementary file 3 — Supplementary Material 3 [file 12913_2024_11222_MOESM3_ESM.docx]

**Additional Table 2.** Risk of bias for observational studies (Newcastle-Ottawa Scale)

|  | **Study design** | **Selection** | | | | **Comparability** | **Outcome** | | | **Total** |
| --- | --- | --- | --- | --- | --- | --- | --- | --- | --- | --- |
|  |  | **1** | **2** | **3** | **4** | **1** | **1** | **2** | **3** |  |
| Agudelo Botero et al. (2013) | Ecological study | * | * | * | * | ** | * | * | * | 9/9 |
| Aguiar et al. (2023) | Cross-sectional study | * | * | * | * | ** | * | * | * | 9/9 |
| de Almeida et al. (2022) | Cross-sectional study | * | * | * | * | ** | * | * | * | 9/9 |
| de Souza et al. (2020) | Ecological study (DATASUS) | * | * | * | * | * | * | * | * | 8/9 |
| Ferreira et al. (2020) | Ecological study (HRC, INCA) | * | * | * | * | * | * | * | * | 8/9 |
| Oliveira et al. (2011) | Ecological study | * | * | * | * | - | * | * | * | 7/9 |
| Saldanha et al. (2019) | Ecological study (DATASUS 2014-2016) | * | * | * | * | - | * | * | * | 7/9 |
| Medeiros et al. (2020) | Prospective cohort study | * | * | * | * | * | * | * | * | 8/9 |
| Recondo et al. (2019) | Prospective cohort study | * | * | * | * | - | * | * | * | 7/9 |
| Rodrigues et al. (2019) | Ecological study | * | - | * | - | ** | * | * | * | 7/9 |
| Romeiro Lopes et al. (2017) | Cross-sectional study | * | * | * | * | ** | * | * | * | 9/9 |
| Gonçalves et al. (2014) | Cross-sectional study | - | - | * | - | - | - | * | * | 3/9 |
| Sousa et al. (2019) | Cross-sectional study | * | - | * | - | ** | * | * | * | 7/9 |
| Amaral et al. (2017) | Cross-sectional study | * | - | * | - | ** | * | * | * | 7/9 |

The scale has been adapted from the Newcastle-Ottawa Quality Assessment Scale for cohort studies to provide quality assessment of cross-sectional studies. For these studies, the principal factor for comparability domain should be the comparability of subjects in different outcome groups on the basis of design or analysis and/or confounding factors controlled. Selection: 1) Representativeness of the exposed cohort, 2) Selection of the non-exposed cohort, 3) Ascertainment of exposure, and 4) Demonstration that outcome of interest was not present at start of study; Comparability: 1) Comparability of cohorts on the basis of the design or analysis; Outcome: 1) Assessment of outcome, 2) Was follow-up long enough for outcomes to occur, and 3) Adequacy of follow-up of cohorts.
